# Next-Generation High-Throughput Functional Annotation of Microbial Genomes

**DOI:** 10.1128/mBio.01245-16

**Published:** 2016-10-04

**Authors:** Ralph S. Baric, Sean Crosson, Blossom Damania, Samuel I. Miller, Eric J. Rubin

**Affiliations:** a Department of Microbiology and Immunology, University of North Carolina, Chapel Hill, North Carolina, USA; b Department of Biochemistry and Molecular Biology and Department of Microbiology, University of Chicago, Chicago, Illinois, USA; c Department of Microbiology, Department of Immunology, Department of Medicine, Department of Genome Sciences, University of Washington, Seattle, Washington, USA; d Department of Immunology and Infectious Disease, Harvard T.H. Chan School of Public Health, Boston, Massachusetts, USA

## Abstract

Host infection by microbial pathogens cues global changes in microbial and host cell biology that facilitate microbial replication and disease. The complete maps of thousands of bacterial and viral genomes have recently been defined; however, the rate at which physiological or biochemical functions have been assigned to genes has greatly lagged. The National Institute of Allergy and Infectious Diseases (NIAID) addressed this gap by creating functional genomics centers dedicated to developing high-throughput approaches to assign gene function. These centers require broad-based and collaborative research programs to generate and integrate diverse data to achieve a comprehensive understanding of microbial pathogenesis. High-throughput functional genomics can lead to new therapeutics and better understanding of the next generation of emerging pathogens by rapidly defining new general mechanisms by which organisms cause disease and replicate in host tissues and by facilitating the rate at which functional data reach the scientific community.

## INTRODUCTION

**FIG 1:**
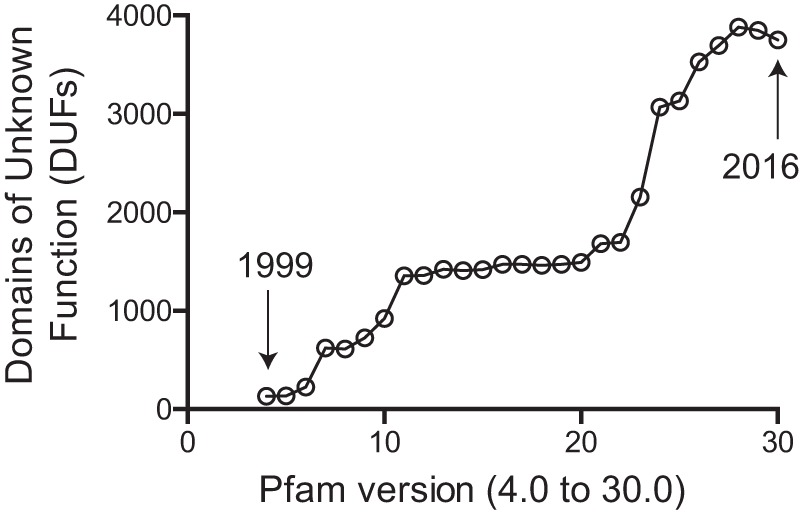
The current release of the protein sequence family database, Pfam (version 30, July 2016; http://pfam.xfam.org/), contains 16,306 sequence family entries, 3,751 of which are families/domains of unknown function (DUFs). The release years for Pfam version 4.0 and version 30.0 are marked with arrows.

Microbial genome sequencing efforts continue at an increasing rate, resulting in an expanding catalogue of genes of unknown function for important pathogens (Fig. 1). In some cases, up to 40% of the annotated genes in fully sequenced microbial genomes have no known or predicted function. In many cases, these uncharacterized genes are highly conserved and are implicated in the pathogenic process through either their timing of expression or requirement for microbial replication. Such data indicate that these genes execute important, general biological functions. The interpretation of existing and emerging microbial genomics data will require the scientific community to uncover new paradigms hidden within these sequences. This point is highlighted by a recent publication by Hutchison et al. focused on building a minimal genome of *Mycoplasma mycoides* (1). The 473 genes in this genome include many required for known essential functions, such as DNA replication, transcription, and translation, but the genome also contains 149 genes (~31%) of unknown function. Likewise, the known coding capacity of DNA viruses has been doubled over the past few years, an achievement made possible by using novel high-throughput sequencing and proteomic methods. The discovery of these genes of unknown function not only promises to affect our understanding of microbial pathogenesis but also provides an undiscovered wealth of new therapeutic targets for antibiotic, antiviral, and vaccine development for improved global and economic health.

Unlike large-scale genome-sequencing or structural-genomics efforts, the functional annotation of uncharacterized genes is not well developed technologically, and therefore, the scientific community cannot rely on a well-defined, mature set of experimental approaches. Simple deletion or overexpression of an uncharacterized gene often fails to yield any discernible phenotype in standard laboratory contexts. In the same vein, a purely biochemical approach that relies on purification and *in vitro* biochemical characterization of uncharacterized genes often fails to yield fruitful functional data. Furthermore, while sequence-based bioinformatic analyses and computational models may provide clues about functional genetic interactions, such predictions are often limited by our current biological knowledge and databases. As a consequence, functional annotation is incorrect in many cases because unvalidated information is propagated across species. In cases where gene function is experimentally assigned and validated, the information is often not broadly propagated or may not apply to another organism. Therefore, a successful functional annotation endeavor requires a multipronged approach that involves open collaboration among scientists with expertise in genetics, bioinformatics, molecular biology, biochemistry, cell physiology, host-microbe interactions, and data management. Such a program is outside the scope of traditional funding mechanisms and requires integrated teams of experts and new high-throughput experimental methodologies.

The NIAID has implemented a program aimed at assigning functions to open reading frames (ORFs) and small noncoding RNAs (ncRNAs) that have been discovered by large-scale sequencing efforts to begin addressing this gap in our understanding of bacterial and viral gene function. This timely initiative from the NIAID is highly significant because it is the institute’s first attempt to incorporate functional annotation into its genomics and advanced technologies program, which is focused on developing genomics, proteomics, and bioinformatics resources to advance our understanding of infectious and immune-mediated diseases. The NIAID Functional Genomics (FG) program builds on previous functional annotation efforts, such as COMBREX (2), and brings together multidisciplinary groups to work on shared goals. Its mission is to probe new biology in pathogenic bacteria and viruses by assigning functions to new genes. The FG program is unique in its approach in that it enables principal investigators to follow up on “omics” and phenotypic screening data with targeted experiments to establish gene function. Most pathogens are evolving constantly, and such an integrated approach will provide the database to allow us to work toward predicting new emerging infectious diseases and respond to new pandemics.

Projects across the NIAID FG program are driven by the idea that the assignment of gene function must be rooted in experimentation *in vivo* and *in vitro*. The challenge to the experimentalist is to develop systematic approaches to gene function discovery that have a reasonable chance of success over a large number of genes. FG Centers are taking a variety of approaches to uncover the functions of protein-coding genes and small ncRNAs. Some approaches, which perhaps will have the highest yield, require at least some prediction of function, while others presuppose no specific functional knowledge. Current state-of-the-art technologies, such as genome synthesis, next-generation nucleic acid sequencing, ribosomal profiling, high-resolution mass spectrometry, metabolomics, and molecular and systems modeling, enable high-throughput approaches to characterize genes of unknown function. Though these technologies are clearly valuable, they are most effectively leveraged when combined with functional assays, such as measurements of microbial permeability and antimicrobial susceptibility or assays of host response to infection, which have been scaled up through recent developments in robotics, whole-genome synthesis, and high-content cellular imaging. In this commentary, we provide an overview of our coordinated efforts that are aimed at filling the gaps in our understanding of microbial gene function. These efforts are restricted to a few organisms and technologies but could be attempted on a much larger scale. Such functional analyses could provide the scientific basis for rational approaches to the development of new therapeutic products to combat future and current gaps in the treatment of bacterial and viral infection.

## *IN VITRO* AND BIOINFORMATIC APPROACHES

### Improving the quality of genome maps.

Most protein-coding genes are annotated by either their similarity to known genes or generalized guidelines for identifying translational start sites. Such predictions are often incorrect. For example, we have demonstrated that a protein highly homologous to a secreted bacterial amidase is actually an intracellular regulator of peptidoglycan synthesis (3). Moreover, predictive algorithms to define the locations of non-protein-coding genes (e.g., small ncRNAs, out-of-frame ORFs, etc.) are generally poor. Defining the boundaries of genes of unknown function is an important component of experimental functional annotation. We are using combinations of directed biochemical, computational, and next-generation-sequencing approaches to produce improved maps of gene boundaries in bacterial and viral pathogens. These efforts enable improved functional genetic and biochemical experiments.

### Leveraging existing protein structure data.

Molecular structure data can provide tremendous insight into the biochemical functions of proteins. A high-quality structural model enables one to map conserved residues on the molecule and to develop hypotheses regarding active-site chemistry, ligand binding sites, protein docking sites, etc. We are taking advantage of the large amount of structural data available in the PDB to build homology-based protein models. In addition, we are using hidden Markov Model (HMM)-based approaches to build predictive structural models in cases where homology in the PDB is low. Experimental structures and high-confidence structural models generated by the FG program are available to the community and have been used to develop and test specific functional hypotheses *in vitro* and *in vivo* and to define protein function (4).

### High-throughput protein production for functional biochemical analysis.

Structural models of proteins of unknown function are informing functional biochemical hypotheses that are being tested *in vitro*. Specifically, we are leveraging existing high-throughput structural genomics infrastructure to produce expression clones of proteins of unknown function. This approach is yielding milligram quantities of many targets of unknown function, which are being assayed for a range of biochemical activities and are being used to produce antibodies for cellular studies. One specific use for these purified proteins is in activity-based metabolomic profiling (5), an unbiased approach to finding substrates and products for putative enzymes.

### Defining gene function by biochemical association.

A well-established approach to define function is to test for physical associations of proteins and RNAs with other proteins or transcripts of known function. We are employing such biochemical association strategies. For protein-coding genes, we are measuring protein-protein associations using quantitative proteomic methods. For example, using a proteomics approach, we reported that Kaposi’s sarcoma-associated herpesvirus (KSHV) viral interferon regulatory factor 1 (vIRF1) can bind the cellular interferon-stimulated gene 15 (ISG15) E3 ligase, HERC5. Interaction of vIRF1 with HERC5 inhibits the conjugation of ISG15 to cellular proteins, thereby dampening the IFN response to the virus (6). Using a functional genomics screen, coupled with synthetic genome design, we demonstrated that the S glycoprotein genes of several severe acute respiratory syndrome coronavirus (SARS-CoV)-like bat coronaviruses can bind human receptors for entry and replicate efficiently in primary human airway epithelial cells and that they are resistant to existing SARS vaccines and immunotherapeutics (7, 8). Our early results suggest that an efficient way to discover function using this approach is to target protein complexes of known function and define associated proteins of unknown function. These efforts have required us to develop new bioinformatics methods to understand the complex data produced by these experiments.

### Prediction of ncRNA targets.

ncRNAs represent a particular challenge in bacterial and virus research, as they can vary tremendously among different bacterial species and viruses and little is known about their functions. We have generated catalogues of the ncRNAs for the organisms (for example, in mycobacteria) (9) that we study and are applying large-scale methods to identify their targets. These methods start with bioinformatics, though the predictive algorithms are not yet particularly robust. In addition, we are applying cross-linking-, ligation-, and sequencing-based approaches to systematically link small ncRNAs of unknown function to their transcript targets. Thus, while many of our approaches to investigating ncRNAs are still at the developmental stage, defining catalogues of ncRNAs provides the community with a road map of targets for downstream studies and analyses.

We are also using next-generation-sequencing technologies, such as transcriptome sequencing (RNAseq) and selective 2′-hydroxyl acylation analyzed by primer extension (SHAPE) analysis of RNA, to investigate RNA structure-function correlations. Paired with these analyses, we are investigating the RNA transcriptome to correlate conserved and unique RNA structure elements with pathogen virulence factors, identify previously uncharacterized and/or rare translation initiation sites, and associate protein structure elements with virus biology, pathogenesis, and host range.

## CELLULAR AND *IN VIVO* APPROACHES

### Genetic approaches: identifying phenotypes.

We are using multiple strategies to identify phenotypes that are associated with the deletion or overexpression of target genes of unknown function. For bacterial pathogens, we have initially focused on genes that, when disrupted, produce measurable growth phenotypes or alterations in cellular barrier function under specified conditions, including antibiotic treatment. These assays are facilitated by whole-genome-mutant defined mutant libraries that allow more efficient high-throughput screening for specific phenotypes (10).

We are assaying the growth of mutant strains in axenic culture and in cell and animal infection models. The Biolog screening platform provides a high-throughput approach to phenotype identification. We are growing individual deletion strains in parallel with wild-type control strains under ~2,000 defined conditions. Differences between strains identify specific medium conditions under which a particular gene is required for wild-type growth and have provided clues for target gene function (11). In a particular case, a functional genetic study of mutants harboring deletions of genes of unknown function is informing the development of a new live attenuated *Brucella abortus* vaccine strain (12). In addition, we are applying genome-wide screening approaches to find genes that interact genetically to modify phenotypes.

In our studies of viral pathogens, we have developed a high-throughput, multiarmed screening program to investigate both RNA and DNA virus-encoded host evasion functions. To accomplish this, we are applying modular cloning technology to easily shuttle candidate genes of unknown function into replicon expression and/or lentiviral vectors for alternate applications, such as the expression of toxic or otherwise challenging proteins and antibody generation. Using these screening assays, we are defining viral counterdefense mechanisms mediated by known, novel, and noncanonical viral ORFs and ncRNAs, whose products modulate the host innate immune response and cellular defense machinery to the virus’s advantage. Screening assays include assessments of beta interferon (IFN-β) antagonism, NF-κB, Toll-like receptor, and apoptosis modulation, inflammasome signaling, cGAS-STING and p53 pathway modulation, cellular localization, global protein synthesis, and mTOR inactivation. For example, we have reported multiple herpesviral proteins that modulate the cGAS-STING DNA sensing pathway and Middle East respiratory syndrome coronavirus (MERS-CoV) and closely related bat MERS-like virus phosphodiesterase proteins that antagonize RNase L activation during infection (13–15). In parallel, we also seek to identify viral entry proteins that program efficient infections across multiple species (7, 8). Our synthetic approach allows us to rapidly test hypothetical proteins—proteins whose expression has not yet been verified in the context of infection. Genes of interest are then deleted or mutated using reverse genetic platforms, and virus mutant growth is examined both in primary human targets, such as airway epithelial cells and various immune cells, and during infection.

## SYNERGIES AND DATA SHARING

### Synergies across the four centers funded by the FG program.

Although the four centers funded by the FG program have different specific goals, we share approaches and are experiencing common challenges, which we are working to solve together. Shared solutions drive research forward. For example, each center includes a small RNA (sRNA) discovery project. There is no generally agreed upon approach to elucidating sRNA gene function. It has been useful to compare experiences and potential solutions across centers. sRNA discovery projects as a group are converging on approaches that may work generally in bacteria. In addition, the data management groups at the four centers share common priorities for public dissemination of data and were able as a group to begin the process of defining new priorities for updating capabilities for the PATRIC and VIPR BRC public resources. An example of this collaboration is the specification of the mode and format for transfer of transposon-sequencing (Tn-Seq) experimental data and Biolog phenotyping data to PATRIC, along with the computational tools to analyze these data (16). This effort will standardize the process of data transfer from independent centers and will define the format for public display of these and other data sets generated by FG Centers.

Another interaction is occurring with the Seattle Structural Genomics Center for Infectious Diseases and the Center for Structural Genomics of Infectious Diseases. Using validated overexpression platforms and novel uncharacterized and/or hypothetical ORFs, the collaboration is designed to determine the structures of high-priority candidate proteins that antagonize host antimicrobial defense pathways in the host. It is likely in the future that the data we generate will be used by the systems biology centers to further create more complex models of host-pathogen interactions.

## CONCLUSIONS

As described above, we have used a multipronged investigation strategy to directly evaluate unknown and hypothetical genes from a diverse array of pathogens to characterize the biological functions encoded by these genes (Table 1). A particular strength of this approach is that this systematic workflow, which can be adapted to all pathogens with sequenced genomes, can ensure rational, directed, and rapid response times for vaccine and therapeutic design in answer to emerging and reemerging epidemics. The work of this program is defining a future blueprint to perform functional analysis of new pathogens as they emerge and to more rapidly respond to the need for knowledge of emerging organisms.

**TABLE 1:** Bacteria and viruses investigated in the NIAID functional genomics program

Bacteria
*Acinetobacter baumannii*
*Mycobacterium tuberculosis* (and related mycobacteria)
*Brucella abortus*
*Yersinia pestis*
Viruses
SARS
MERS
Influenza
Ebola
KSHV
Zika

## References

[1] Hutchison CA III, Chuang RY, Noskov VN, Assad-Garcia N, Deerinck TJ, Ellisman MH, Gill J, Kannan K, Karas BJ, Ma L, Pelletier JF, Qi ZQ, Richter RA, Strychalski EA, Sun L, Suzuki Y, Tsvetanova B, Wise KS, Smith HO, Glass JI, Merryman C, Gibson DG, Venter JC 2016 Design and synthesis of a minimal bacterial genome. Science 351:aad6253. doi:10.1126/science.aad6253. 27013737

[2] Anton BP, Chang Y-C, Brown P, Choi H-P, Faller LL, Guleria J, Hu Z, Klitgord N, Levy-Moonshine A, Maksad A, Mazumdar V, McGettrick M, Osmani L, Pokrzywa R, Rachlin J, Swaminathan R, Allen B, Housman G, Monahan C, Rochussen K, Tao K, Bhagwat AS, Brenner SE, Columbus L, de Crecy-Lagard V, Ferguson D, Fomenkov A, Gadda G, Morgan RD, Osterman AL, Rodionov DA, Rodionova IA, Rudd KE, Soll D, Spain J, Xu SY, Bateman A, Blumenthal RM, Bollinger JM, Chang WS, Ferrer M, Friedberg I, Galperin MY, Gobeill J, Haft D, Hunt J, Karp P, Klimke W, Krebs C, Macelis D, et al. 2013 The COMBREX project: design, methodology, and initial results. PLoS Biol 11:e1001638. doi:10.1371/journal.pbio.1001638. 24013487 PMC3754883

[3] Boutte CC, Baer CE, Papavinasasundaram K, Liu W, Chase MR, Meniche X, Fortune SM, Sassetti CM, Ioerger TR, Rubin EJ 2016 A cytoplasmic peptidoglycan amidase homologue controls mycobacterial cell wall synthesis. Elife 5:e14590. doi:10.7554/eLife.14590. 27304077 PMC4946905

[4] Willett JW, Herrou J, Briegel A, Rotskoff G, Crosson S 2015 Structural asymmetry in a conserved signaling system that regulates division, replication, and virulence of an intracellular pathogen. Proc Natl Acad Sci U S A 112:E3709–E3718. doi:10.1073/pnas.1503118112. 26124143 PMC4507200

[5] de Carvalho LP, Zhao H, Dickinson CE, Arango NM, Lima CD, Fischer SM, Ouerfelli O, Nathan C, Rhee KY 2010 Activity-based metabolomic profiling of enzymatic function: identification of Rv1248c as a mycobacterial 2-hydroxy-3-oxoadipate synthase. Chem Biol 17:323–332. doi:10.1016/j.chembiol.2010.03.009. 20416504 PMC2878197

[6] Jacobs SR, Stopford CM, West JA, Bennett CL, Giffin L, Damania B 2015 Kaposi’s sarcoma-associated herpesvirus viral interferon regulatory factor 1 interacts with a member of the interferon-stimulated gene 15 pathway. J Virol 89:11572–11583. doi:10.1128/JVI.01482-15. 26355087 PMC4645652

[7] Menachery VD, Yount BL Jr, Debbink K, Agnihothram S, Gralinski LE, Plante JA, Graham RL, Scobey T, Ge XY, Donaldson EF, Randell SH, Lanzavecchia A, Marasco WA, Shi ZL, Baric RS 2015 A SARS-like cluster of circulating bat coronaviruses shows potential for human emergence. Nat Med 21:1508–1513. doi:10.1038/nm.3985. 26552008 PMC4797993

[8] Menachery VD, Yount BL Jr, Sims AC, Debbink K, Agnihothram SS, Gralinski LE, Graham RL, Scobey T, Plante JA, Royal SR, Swanstrom J, Sheahan TP, Pickles RJ, Corti D, Randell SH, Lanzavecchia A, Marasco WA, Baric RS 2016 SARS-like WIV1-CoV poised for human emergence. Proc Natl Acad Sci U S A 113:3048–3053. doi:10.1073/pnas.1517719113. 26976607 PMC4801244

[9] Shell SS, Wang J, Lapierre P, Mir M, Chase MR, Pyle MM, Gawande R, Ahmad R, Sarracino DA, Ioerger TR, Fortune SM, Derbyshire KM, Wade JT, Gray TA 2015 Leaderless transcripts and small proteins are common features of the mycobacterial translational landscape PLoS Genet 11:e1005641. doi:10.1371/journal.pgen.1005641. 26536359 PMC4633059

[10] Gallagher LA, Ramage E, Weiss EJ, Radey M, Hayden HS, Held KG, Huse HK, Zurawski DV, Brittnacher MJ, Manoil C 2015 Resources for genetic and genomic analysis of emerging pathogen Acinetobacter baumannii. J Bacteriol 197:2027–2035. doi:10.1128/JB.00131-15. 25845845 PMC4438207

[11] Herrou J, Czyż DM, Willett JW, Kim HS, Chhor G, Babnigg G, Kim Y, Crosson S 2016 WrpA is an atypical flavodoxin family protein under regulatory control of the Brucella abortus general stress response system. J Bacteriol 198:1281–1293. doi:10.1128/JB.00982-15. 26858101 PMC4859584

[12] Willett JW, Herrou J, Czyz DM, Crosson S 2016 *Brucella abortus* Δ*rpoE1* confers protective immunity against wild type challenge in a mouse model of brucellosis. Vaccine 34:5073–5081. doi:10.1016/j.vaccine.2016.08.076. 27591954 PMC5026968

[13] Ma Z, Jacobs SR, West JA, Stopford C, Zhang Z, Davis Z, Barber GN, Glaunsinger BA, Dittmer DP, Damania B 2015 Modulation of the cGAS-STING DNA sensing pathway by gammaherpesviruses. Proc Natl Acad Sci U S A 112:E4306–E4315. doi:10.1073/pnas.1503831112. 26199418 PMC4534226

[14] Sun C, Schattgen SA, Pisitkun P, Jorgensen JP, Hilterbrand AT, Wang LJ, West JA, Hansen K, Horan KA, Jakobsen MR, O’Hare P, Adler H, Sun R, Ploegh HL, Damania B, Upton JW, Fitzgerald KA, Paludan SR 2015 Evasion of innate cytosolic DNA sensing by a gammaherpesvirus facilitates establishment of latent infection. J Immunol 194:1819–1831. doi:10.4049/jimmunol.1402495. 25595793 PMC4323864

[15] Thornbrough JM, Jha BK, Yount B, Goldstein SA, Li Y, Elliott R, Sims AC, Baric RS, Silverman RH, Weiss SR 2016 Middle East respiratory syndrome coronavirus NS4b protein inhibits host RNase L activation. mBio 7:e00258. doi:10.1128/mBio.00258-16. 27025250 PMC4817253

[16] DeJesus MA, Ambadipudi C, Baker R, Sassetti C, Ioerger TR 2015 TRANSIT—a software tool for Himar1 TnSeq analysis. PLoS Comput Biol 11:e1004401. doi:10.1371/journal.pcbi.1004401. 26447887 PMC4598096

